# Influence of low birth weight on C-reactive protein in asymptomatic younger adults: the bogalusa heart study

**DOI:** 10.1186/1756-0500-4-71

**Published:** 2011-03-21

**Authors:** Azad R Bhuiyan, Sathanur R Srinivasan, Wei Chen, Mario J Azevedo, Gerald S Berenson

**Affiliations:** 1 Department of Epidemiology and Biostatistics, Jackson State University, MS, USA; 2 Tulane Center for Cardiovascular Health, Tulane University Health Sciences Center, LA, USA

## Abstract

**Background:**

Both low birth weight, an indicator of intrauterine growth restriction, and low grade systemic inflammation depicted by high sensitivity C-reactive protein (hs-CRP) have emerged as independent predictors of cardiovascular (CV) disease and type 2 diabetes. However, information linking low birth weight and hs-CRP in a biracial (black/white) population is scant. We assessed a cohort of 776 black and white subjects (28% black, 43% male) aged 24-43 years (mean 36.1 years) enrolled in the Bogalusa Heart Study with regard to birth weight and gestational age data were retrieved from Louisiana State Public Health Office.

**Findings:**

Black subjects had significantly lower birth weight than white subjects (3.145 kg vs 3.441 kg, p < 0.0001) and higher hs-CRP level (3.29 mg/L vs 2.57 mg/L, p = 0.011). After adjusting for sex, age, body mass index (BMI), smoking status and race (for total sample), the hs-CRP level decreased across quartiles of increasing birth weight in white subjects (p = 0.001) and the combined sample (p = 0.002). Adjusting for sex, age, BMI, smoking status and race for the total sample in a multivariate regression model, low birth weight was retained as an independent predictor variable for higher hs-CRP levels in white subjects (p = 0.004) and the total sample (p = 0.007). Conversely, the area under the receiver operative curve (c statistic) analysis adjusted for race, sex, age, smoking status and BMI yielded a value of 0.777 with regard to the discriminating value of hs-CRP for predicting low birth weight.

**Conclusions:**

The deleterious effect of low birth weight on systemic inflammation depicted by the hs-CRP levels in asymptomatic younger adults may potentially link fetal growth retardation, CV disease and diabetes, with important health implications.

## Background

The growth of an undernourished fetus results in adaptive fetal programming or metabolic imprinting, with permanent changes in structure, metabolism and physiology of fetal organs and related pathophysiologic consequences in later life [1,2]. Studies worldwide, regardless of socio-economic background, have linked low birth weight to cardiometabolic risk factors, related cardiovascular (CV) disease, and type 2 diabetes [3,4]. Recently, we reported the adverse relationship of low birth weight to white blood cell count and pulsatile arterial function [5,6].

Inflammation plays an important role in the pathogenesis of atherosclerosis [7-9]. Risk factors, e.g. cigarette smoking, hypertension, dyslipidemia and hyperglycemia promote inflammation, are well established. Biomarkers, including as oxidized low-density lipoproteins, interleukin-6, intercellular adhesion molecule-1 and high sensitivity C-reactive protein (hs-CRP, reflect the ongoing inflammatory process [8]. Of these, hs-CRP - an acute-phase reactant secreted by the liver - has emerged as an independent predictor of CV disease and type 2 diabetes [8-13]. In terms of birth weight, a few studies have demonstrated an inverse association between birth weight and hs-CRP in children and adults [14-16]. Consequently, the American Heart Association and Center for Disease Control recommended guidelines for the incorporation of hs-CRP into its CV disease risk stratification [8]. However, population-based data in this regard are scant. The present study examines this aspect in younger adults enrolled in the Bogalusa Heart Study, a biracial (35% black/65% white) community-based investigation of the natural history of CV disease

## Materials and methods

### Study Population

The study sample was derived from a cohort of 1203 subjects aged 23 to 43 examined as a part of a longitudinal follow-up survey. Of these, information on 908 singletons with hs-CRP measurement, birth weight, gestational age and body mass index (BMI) data were available. The exclusion of 132 singletons born premature (<37 weeks of gestation) and/or had conditions (with or without medication) of diabetes, hypertension and dyslipidemia left 776 eligible participants (28% black, 43% male, mean age 36.1 years). Birth weight and gestational age data were retrieved from Louisiana State Public Health Office. Tulane University Medical Center Institutional Review Board approved the study, and informed consent was obtained from all participants.

### Measurements

Standardized techniques and protocols were used by trained examiners. Height and weight were measured twice and the mean values were used to calculate BMI as a measure of adiposity. Information on smoking status was obtained by questionnaires. Those who had smoked at least one cigarette per week during the past one year or more were identified as current smokers, and the remainder as non-smokers. Plasma high sensitivity hs-CRP levels were measured by latex particle-enhanced immunoturbidemetric assay on a Hitachi 902 Automatic Analyzer (Roche Diagnostics, Indianapolis, IN, USA). The reproducibility of hs-CRP measurement checked with 10% of randomly assigned pairs of blind duplicate analysis, which gave an intraclass correlation coefficient of 0.99.

### Statistical Methods

Statistical analyses were done with SAS software, version 9.1 (SAS, Carey, NC). As the hs-CRP level was not normally distributed, log transformation was used to approach normality. Analyses were performed where appropriate on transformed data. Analysis of variance for race difference in mean values of continuous variables and chi-square test for categorical variables were used. Because of a strong association between gestational age and birth weight, the latter was adjusted by regression analysis models to the mean values of the former in each race-sex group. For categorical analysis, quartiles of gestational age-adjusted birth weight were defined by using cut-off points in race-sex groups. Covariates adjusted mean values of hs-CRP were calculated by general linear models and used for analysis of birth weight by quartiles. Multiple regression analysis was used to determine the independent association of birth weight with hs-CRP in relation to the measured covariates. Two steps were followed in the regression model: the logarithm of hs-CRP was used in both cases as a dependent variable to determine the association between birth weight and hs-CRP. Model 1 was adjusted for age, sex, smoking and race (for the total sample) and the model 2 was adjusted for the above variables along with the potential confounding variable, BMI. In addition, a multivariate c-statistic model was used to determine the ability of hs-CRP to predict low birth weight (defined as <2500 gm). The area under the receiver-operating characteristic curve (c-statistic) was evaluated after adjusting for the covariates race, sex, age, smoking status and BMI. C-statistic of >0.5 indicated increased predictive ability.

## Results

Table 1 shows the mean values of hs-CRP, birth weight, and other study variables of younger adults by race. Black subjects had significantly lower gestational age and birth weight, but higher BMI and hs-CRP levels, than white subjects.

**Table 1:** Birth Weight, hs-CRP and other study variables of young adults by race

	White (n = 559)	Black (n = 217)	p-value^b^
Age (year)^a^	36.3 (4.3)	35.8 (4.7)	0.200
Males/Females (%)	46/54	35/65	-------
BMI (kg/m^2^)^a^	28.3 (6.3)	30.5 (7.8)	<0.0001
Gestational age (week)^a^	40.1 (1.2)	39.8 (1.0)	0.001
Birth weight (kg)^a^	3.441 (0.48)	3.154 (0.47)	<0.0001
hs-CRP (mg/L)^a^	2.57 (3.1)	3.29 (3.5)	0.011
Smoker (%)	44.1	40.3	0.327

^a ^Mean (SD)

^b ^p value for race difference

**Figure 1:**
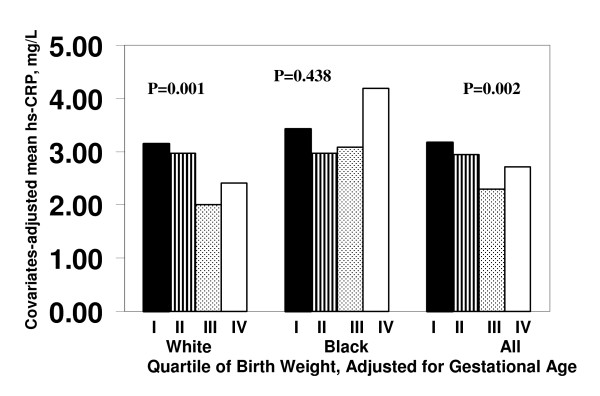
**Covariates-adjusted mean values of hs-CRP by race and sex-specific quartiles of gestational age-adjusted birth weight: The Bogalusa Heart Study**. Quartile I represents the lowest birth weight, and quartile IV represents the highest birth weight. Covariates included race (for the total sample), sex, age, body mass index and smoking status.

Figure 1 illustrates the relation of covariates-adjusted mean levels of hs-CRP to race- and sex-specific quartiles of gestational age-adjusted birth weight. The covariates included race (for the total sample), sex, age, BMI and smoking status. The hs-CRP level significantly decreased with increasing quartiles of birth weight among white subjects (p = 0.001) and the total sample (p = 0.002). Black subjects showed no such significant trend.

Table 2 presents the multivariate linear regression analysis of hs-CRP on gestational age-adjusted birth weight and other covariates in white subjects, black subjects and the total sample. Birth weight was independently and inversely associated with hs-CRP in white subjects in models without or with BMI as a covariate. In the total sample, such independent inverse association was noted only in model 2 that included BMI as a covariate. Also, sex (females > males), BMI (positive association), and smoking (positive association) were independently correlated with hs-CRP in white subjects and the total sample in model 2.

**Table 2:** Predictors of hs-CRP in young adults by race.

	White		Black		All	
	ß	p-value	ß	p-value	ß	p-value
**Model 1**						
Black race	----	------	------	------	0.18	0.08
Age (year)	-0.0005	0.97	0.03	0.10	0.008	0.41
Female sex	0.42	<0.0001	0.38	0.04	0.41	<0.0001
Smoking (yes)	0.15	0.14	-0.35	0.05	0.01	0.87
Birth weight (kg)^a^	-0.22	0.05	0.03	0.67	-0.14	0.13
**Model 2**						
Black race	----	----	-----	-----	-0.01	0.90
Age (year)	0.01	0.26	0.02	0.25	0.01	0.15
Female sex	0.50	<0.0001	0.26	0.12	0.43	<0.0001
Smoking (yes) Birth weight (kg)^a^	0.30 -0.28	0.002 0.004	-0.12 -0.13	0.48 0.47	0.19 -0.23	0.02 0.007
BMI (kg/m^2^)	0.08	<0.0001	0.07	<0.0001	0.08	<0.0001

The Bogalusa Heart Study.

^a ^Gestational age-adjusted birth weight

**Figure 2:**
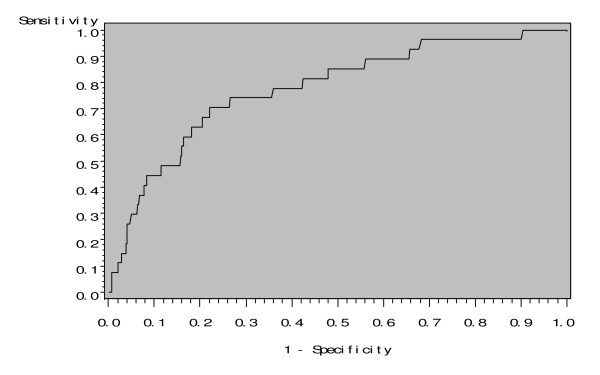
**The area under the receiver-operating curve of hs-CRP for predicting low birth weight, adjusted for race, sex, age, smoking status and body mass index**. The c-statistic was 0.777.

Figure 2 shows the discriminative value of hs-CRP for associating with low birth weight, after adjusting for race, sex, age, smoking status and BMI. The c-value for hs-CRP was 0.777.

## Discussion

This community-based study demonstrates an inverse and independent association between birth weight and hs-CRP, a widely used biomarker of systemic inflammation. In addition, female sex, BMI and smoking were independent adverse correlates of hs-CRP. It is noteworthy that these findings support the emerging concept of intrauterine imprinting and its pathophysiologic consequences later in life by linking low birth weight to excess hs-CRP [1,2].

Although previous study in children has failed to find significant association between birth weight and hs-CRP [16], our epidemiologic study is consistent with the MIDSPAN family study and Northern Finland 1966 birth cohort study in adults [14,15]. However, this inverse relationship was not observed in black subjects in our analysis. Of note, black subjects had significantly lower birth weights and related higher hs-CRP levels than white subjects [17,18].

Although observational studies like this cannot address the issue of causality, several putative mechanisms might link low birth weight to underlying perturbation in inflammatory pathways in utero. Undernutrition in utero causes permanent impairment in growth, structure, and function of muscle [19,20], fat [21,22], liver [23] and renal nephrons [9,24] inter alia due to adaptive programming, resulting in cardiometabolic syndrome and related disorders in later life [3,25]. Interestingly, in utero muscle growth is retarded in low-birth weight babies [25]; and since there is little muscle cell replication after birth [26], these individuals will develop a disproportionately high fat mass and related state of chronic low-grade inflammation induced by adipose tissue cells including monocytes in a nutritionally-rich environment of postnatal life [25,27]. Acute-phase reactants secreted by the liver, including hs-CRP, are upregulated by interleukin-6, a proinflammatory cytokine synthesized by adipose tissue and cleared mainly by the kidneys. Hence, adaptive programming of any of these tissues may result in heightened inflammatory states later in life. Of note, Barker et al. [28] demonstrated an inverse relationship between birth weight and acute-phase reactant fibrinogen in adults. Furthermore, studies including our own have shown an inverse relationship between birth weight and the number of circulating leukocytes [5,29], the cellular effectors of inflammation. However, a caveat relating to causality, is that the observed elevation in hs-CRP levels among low birth weight adults may be a reverse causation phenomenon secondary to underlying excess atherosclerosis [30,31].

As in previous studies [18,32], obesity measured as BMI has been identified as an independent correlate of hs-CRP in our cohort. As discussed above, this is consistent with the pathophysiological role of adipose tissue in regulating inflammation. Furthermore, sex, but not race, was retained as an independent correlate of hs-CRP in our subjects. The excess in hs-CRP in females, also noted in previous study [18], may be due to an estrogen effect [33]. Based on exogenous estrogen administration studies in women, this hormone has been implicated in the transcriptional control, clearance, or cytokine regulation of several acute-phase reactants produced by the liver, including hs-CRP [34], but the role of endogenous estrogen in this regard is unknown. The current observations on white and total subjects also support the known adverse influence of smoking behavior on hs-CRP levels [35]. The lack of adverse effect of smoking among black subjects may be due to differences in intensity and duration of smoking.

In conclusion, low birth weight for gestational age is characterized by increased hs-CRP levels. In conjunction with earlier studies, these findings support the view that low birth weight, albeit a crude surrogate indicator of adaptive fetal programming, is a potential early risk factor for the emergence of disorders related to the activation of inflammatory pathways. As stated by Barker [1], primary prevention lies in protecting fetal development.

## Abbreviations

hs-CRP: high sensitivity C-reactive protein; BMI: body mass index.

## Competing interests

The authors declare that they have no competing interests.

## Authors' contributions

ARB participated in study design, data analysis and manuscript preparation. SRB, WC and GSB contributed to study concept and design, data collection, acquisition of funding and manuscript preparation. MJA involved in manuscript preparation. All authors listed have made substantial contributions on this article

## References

[B1] Barker DJ Fetal Origins of Cardiovascular and Lung Disease 2001 New York, NY: Marcel Dekker, Inc 97 197

[B2] Waterland RA Garza C Potential mechanisms of metabolic imprinting that lead to chronic disease Am J Clin Nutr 1999 69 179 197 9989679 10.1093/ajcn/69.2.179

[B3] Barker DJ Hales CN Fall CH Osmond C Phipps K Clark PM Type 2 (non-insulin-dependent) diabetes mellitus, hypertension and hyperlipidaemia(syndrome X): relation to reduced fetal growth Diabetologia 1993 36 62 67 10.1007/BF00399095 8436255

[B4] Leon DA Lithell HO Vâgerö D Koupilová I Mohsen R Berglund L Lithell UB McKeigue PM Reduced fetal growth rate and increased risk of death from ischaemic heart disease: cohort study of 15 000 Swedish men and women born 1915-29 BMJ 1998 317 241 245 9677213 10.1136/bmj.317.7153.241 PMC28614

[B5] Chen W Srinivasan SR Berenson GS Influence of birth weight on white blood cell count in biracial (black-white) children, adolescents, and young adults: the Bogalusa Heart Study Am J Epidemiol 2009 169 214 218 10.1093/aje/kwn341 19064646 PMC2727256

[B6] Bhuiyan AR Chen W Srinivasan SR Azevedo MJ Berenson GS Relationship of low birth weight to pulsatile arterial function in asymptomatic younger adults: the Bogalusa Heart Study Am J Hypertens 2010 23 168 173 10.1038/ajh.2009.218 19942864

[B7] Ross R Atherosclerosis: an inflammatory disease N Engl J Med 1999 340 115 125 10.1056/NEJM199901143400207 9887164

[B8] Pearson TA Mensah GA Alexander RW Markers of inflammation and cardiovascular disease. Application to clinical and public health practice: a statement for healthcare professionals from the Centers for Disease Control and Prevention and the American Heart Association Circulation 2003 107 499 511 10.1161/01.CIR.0000052939.59093.45 12551878

[B9] Grundy SM Inflammation, hypertension, and the metabolic syndrome JAMA 2003 290 3000 3002 10.1001/jama.290.22.3000 14665663

[B10] Rawson ES Freedson PS Osganian SK Matthews CE Reed G Ockene IS Body mass index, but not physical activity, is associated with C-reactive protein Med Sci Sports Exerc 2003 35 1160 1166 10.1249/01.MSS.0000074565.79230.AB 12840637

[B11] Wong ND Pio J Valencia R Thakal G Distribution of C-reactive protein and its relation to risk factors and coronary heart disease risk estimation in the National Health and Nutrition Examination Survey (NHANES) III Prev Cardiol 2001 4 109 114 10.1111/j.1520-037X.2001.00570.x 11828186

[B12] Freeman DJ Norrie J Caslake MJ Gaw A Ford I Lowe GD O'Reilly DS Packard CJ Sattar N C-reactive protein is an independent predictor of risk for the development of diabetes in the West of Scotland Coronary Prevention Study Diabetes 2002 51 1596 1600 10.2337/diabetes.51.5.1596 11978661

[B13] Pradhan AD Manson JE Rifai N Buring JE Ridker PM C-reactive protein, interleukin 6, and risk of developing type 2 diabetes mellitus JAMA 2001 286 327 334 10.1001/jama.286.3.327 11466099

[B14] Sattar N McConnachie A O'Reilly D Upton MN Greer IA Davey Smith G Watt G Inverse association between birth weight and C-reactive protein concentrations in the MIDSPAN Family Study Arterioscler Thromb Vasc Biol 2004 24 583 587 10.1161/01.ATV.0000118277.41584.63 14739124

[B15] Tzoulaki I Jarvelin MR Hartikainen AL Leinonen M Pouta A Paldanius M Ruokonen A Canoy D Sovio U Saikku P Elliott P Size at birth, weight gain over the life course, and low-grade inflammation in young adulthood: northern Finland 1966 Birth Cohort study Eur Heart J 2008 29 1049 56 10.1093/eurheartj/ehn105 18403494

[B16] Gillum RF Association of serum C-reactive protein and indices of body fat distribution and overweight in Mexican American children J Natl Med Assoc 2003 95 545 552 12911252 PMC2594647

[B17] Frerichs RR Srinivasan SR Webber LS Rieth MC Berenson GS Serum lipids and lipoproteins at birth in a biracial population: the Bogalusa heart study Pediatr Res 1978 12 858 863 10.1203/00006450-197808000-00011 210440

[B18] Patel DA Srinivasan SR Xu JH Li S Chen W Berenson GS Distribution and metabolic syndrome correlates of plasma C-reactive protein in biracial (black-white) younger adults: the Bogalusa Heart Study Metabolism 2006 55 699 705 10.1016/j.metabol.2005.07.015 16713426

[B19] Phillips DI Insulin resistance as a programmed response to fetal undernutrition Diabetologia 1996 39 1119 22 10.1007/BF00400663 8877298

[B20] Taylor DJ Thompson CH Kemp GJ Barnes PR Sanderson AL Radda GK Phillips DI A relationship between impaired fetal growth and reduced muscle glycolysis revealed by 31P magnetic resonance spectroscopy Diabetologia 1995 38 1205 1212 10.1007/BF00422370 8690173

[B21] Lapillonne A Braillon P Claris O Chatelain PG Delmas PD Salle BL Body composition in appropriate and in small for gestational age infants Acta Paediatr 1997 86 196 200 10.1111/j.1651-2227.1997.tb08868.x 9055893

[B22] Jaquet D Gaboriau A Czernichow P Levy-Marchal C Insulin resistance early in adulthood in subjects born with intrauterine growth retardation J Clin Endocrinol Metab 2000 85 1401 1406 10.1210/jc.85.4.1401 10770173

[B23] Barker DJ Martyn CN Osmond C Wield GA Abnormal liver growth in utero and death from coronary heart disease BMJ 1995 310 703 704 7711538 10.1136/bmj.310.6981.703 PMC2549097

[B24] Hinchliffe SA Lynch MR Sargent PH Howard CV Van Velzen D The effect of intrauterine growth retardation on the development of renal nephrons Br J Obstet Gynaecol 1992 99 296 301 10.1111/j.1471-0528.1992.tb13726.x 1581274

[B25] Eriksson JG Growth and coronary heart disease in adult life CVR & R 2002 23 557 560

[B26] Widdowson EM Crabb DE Milner RD Cellular Development of Some Human Organs Before Birth Arch Dis Child 1972 4 7 652 655 10.1136/adc.47.254.652 PMC1648291 5046781

[B27] Rajala MW Scherer PE Minireview: The adipocyte--at the crossroads of energy homeostasis, inflammation, and atherosclerosis Endocrinology 2003 144 3765 3773 10.1210/en.2003-0580 12933646

[B28] Barker DJ Meade TW Fall CH Lee A Osmond C Phipps K Stirling Y Relation of fetal and infant growth to plasma fibrinogen and factor VII concentrations in adult life BMJ 1992 304 148 152 10.1136/bmj.304.6820.148 1737158 PMC1881173

[B29] Canoy D Pouta A Ruokonen A Hartikainen AL Saikku P Järvelin MR Weight at birth and infancy in relation to adult leukocyte count: a population-based study of 5619 men and women followed from the fetal period to adulthood J Clin Endocrinol Metab 2009 94 1916 1922 10.1210/jc.2008-2545 19276227

[B30] Schunkert H Samani NJ Elevated C-reactive protein in atherosclerosis--chicken or egg? N Engl J Med 2008 359 1953 1955 10.1056/NEJMe0807235 18971498

[B31] Shah SH de Lemos JA Biomarkers and cardiovascular disease: determining causality and quantifying contribution to risk assessment JAMA 2009 302 92 93 10.1001/jama.2009.949 19567447

[B32] Tracy RP Is visceral adiposity the "enemy within"? Arterioscler Thromb Vasc Biol 2001 21 881 883 11397691 10.1161/01.atv.21.6.881

[B33] Rifai N Ridker PM Population distributions of C-reactive protein in apparently healthy men and women in the United States: implication for clinical interpretation Clin Chem 2003 49 666 669 10.1373/49.4.666 12651826

[B34] Ridker PM Hennekens CH Rifai N Buring JE Manson JE Hormone replacement therapy and increased plasma concentration of C-reactive protein Circulation 1999 100 713 716 10449692 10.1161/01.cir.100.7.713

[B35] Bazzano LA He J Muntner P Vupputuri S Whelton PK Relationship between cigarette smoking and novel risk factors for cardiovascular disease in the United States Ann Intern Med 2003 138 891 897 12779299 10.7326/0003-4819-138-11-200306030-00010

